# Prolonged Overtime Predicts Worsening Burnout Among Healthcare Workers: A 4-Year Longitudinal Study in Taiwan

**DOI:** 10.3390/healthcare13151859

**Published:** 2025-07-30

**Authors:** Yong-Hsin Chen, Gwo-Ping Jong, Ching-Wen Yang, Chiu-Hsiang Lee

**Affiliations:** 1 Department of Public Health, Chung Shan Medical University, Taichung 402, Taiwan; a6328539@gmail.com; 2 Department of Occupational Safety and Health, Chung Shan Medical University Hospital, Taichung 402, Taiwan; thesarina1120@gmail.com; 3 Department of Internal Medicine, Chung Shan Medical University Hospital, Taichung 402, Taiwan; cshy1506@csh.org.tw; 4 Institute of Medicine, Chung Shan Medical University, Taichung 402, Taiwan; 5 Department of Nursing, Chung Shan Medical University, Taichung 402, Taiwan; 6 Department of Nursing, Chung Shan Medical University Hospital, Taichung 402, Taiwan

**Keywords:** burnout, overtime, healthcare workers, longitudinal study, survival analysis

## Abstract

**Background:** Overtime adversely affects physical and mental health, contributing to irritability, anxiety, reduced sleep, and even cardiovascular issues, ultimately lowering care quality and increasing turnover intentions. This study aimed to investigate whether prolonged overtime increases the risk of occupational burnout over time among healthcare workers. **Methods:** We conducted a four-year longitudinal observational study using secondary data from annual surveys (2021–2024) of healthcare workers at a medical university hospital in Taichung, Taiwan. Burnout was assessed using the personal burnout (PB) scale from the Copenhagen Burnout Inventory (CBI), with high PB levels (HPBL) defined as scores in the upper quartile of the 2021 baseline. Survival analysis utilizing the Kaplan–Meier method and Cox regression investigated burnout progression and the effects of overtime. **Results:** HPBL was defined as PB scores ≥45.83 (upper quartile in 2021). The proportions of HPBL were 30.28% (2021), 33.29% (2022), 36.75% (2023), and 32.51% (2024). Survival analysis confirmed that the risk of burnout increased over time, with the survival time estimated at 2.50 ± 0.03 years and lower survival probabilities observed among participants working overtime (Log-rank test, *p* < 0.0001). Multivariate logistics revealed overtime work, female gender, being a physician/nurse, and reduced sleep as independent risk factors for HPBL (OR = 3.14 for overtime, *p* < 0.001). These findings support the hypotheses on burnout progression and the impact of overtime. **Conclusions:** Overtime significantly heightens the risk of burnout, which worsens over time. Female sex, healthcare roles, obesity, and insufficient sleep are additional risk factors. Limiting overtime and proactive interventions are crucial to preventing burnout in healthcare workers.

## 1. Introduction

Burnout is a state of exhaustion caused by prolonged exposure to work-related problems [1] or sustained involvement in emotionally demanding work environments [2]. It results from unmanaged chronic stress [3]. A multistage process, burnout develops gradually, starting with initial work enthusiasm, followed by increasing pressure, conflicts, shifts in values, depression, and finally, the emergence of burnout symptoms [4].

Without timely intervention, individuals experiencing burnout may exhibit clinical symptoms such as emotional exhaustion, physical fatigue, cognitive impairment, sleep disturbances, and functional decline [5,6]. In severe cases, burnout can result in depression or anxiety disorders [5]. These effects are particularly concerning in the medical workplace, where they can diminish nurses’ well-being [7], weaken the workforce [8], and lead to significant professional consequences, including decreased patient satisfaction [9], compromised quality of care [10], and increased medical errors [11].

The absence of a clear, unified definition of burnout syndrome has contributed to variations in its reported prevalence, ranging from approximately 4% to 25% across different regions and countries [3].

Healthcare workers are at particularly high risk, with the prevalence of burnout estimated to be between 10% [12] and 50% [13]. This heightened risk stems from the intense stress of close contact with patients and their families. These findings underscore the significant threat that burnout poses to the mental health of healthcare professionals.

In Asian healthcare systems, however, additional contextual factors may further exacerbate burnout risk, including hierarchical organizational culture, gendered labor expectations, and a strong overtime norm. In Taiwan, for instance, physicians are often subject to systemic overtime requirements [14], and nurses routinely work more than 50 h per week [15]. These working conditions are often accepted as normative, with limited institutional mechanisms for stress management or psychological support. Specifically, the term “prolonged overtime” refers to working hours that exceed recognized health or legal thresholds. In Taiwan, the Labor Standards Act sets a monthly overtime limit of 46 h [16], while in Japan, 45 h per month is widely accepted in regulations and research as a critical threshold for adverse health effects [17]. This benchmark not only reflects legal expectations, but also aligns with real-world health risks observed among healthcare professionals. This contrasts with Western contexts, where more formalized work-hour protections and burnout prevention strategies are in place.

Research has consistently shown that overtime or extended working hours harm physical and mental health, resulting in irritability, fatigue, anxiety, depression, somatic symptoms [18], reduced sleep duration, and poorer subjective sleep quality [19]. Extended working hours also increase the risk of cardiovascular diseases [20]. In medical settings, these health impacts can reduce the quality of care [21] and increase the incidence of healthcare-associated infections [22].

Additionally, overtime work is strongly associated with higher staff turnover intentions [23] and increased work–family conflict [24]. These findings highlight the serious harm caused by overtime in medical institutions and underscore the need for urgent attention.

In summary, burnout and overtime significantly impact medical staff’s physical and mental health. Interestingly, the two conditions share many overlapping symptoms. This raises a critical question: Is there a close link between overtime and occupational burnout?

Although this association is widely assumed, prior research on this topic has primarily employed cross-sectional designs, limiting understanding of how burnout risk evolves over time or differs across organizational cultures. Notably, very few longitudinal studies have been conducted within Asian healthcare systems, especially in Taiwan, where high-intensity work environments and structural overtime culture persist.

Research on burnout has primarily involved cross-sectional studies, which do not fully capture the long-term effects of risk factors on burnout. Notably, prior studies have indicated that burnout symptoms—particularly emotional exhaustion and frustration—may accumulate and intensify over time [25], which warrants deeper investigation. To fill this gap, adopting a longitudinal approach that enables us to explore the temporal dynamics of burnout development is crucial.

Moreover, understanding how overtime contributes to the timing and onset of burnout, rather than just its presence, can inform culturally relevant intervention strategies tailored to the Asian medical workforce.

The objective of this study is to examine whether prolonged overtime increases the risk of burnout over time among healthcare workers. Thus, we propose the following hypotheses for further testing:

**H1.**  *The risk of burnout increases over time*.

**H2.**  *Individuals who work overtime face a higher burnout risk*.

By testing these hypotheses, we aim to clarify whether extended overtime directly affects the risk of occupational burnout. This study adds value by applying survival and logistic regression models to a four-year longitudinal dataset from Taiwan, situating the findings within a broader socio-cultural context and offering evidence-based implications for workforce policy. In addition to empirically testing these hypotheses, this study seeks to provide practical insights for early identification and prevention of burnout in healthcare systems, with the aim of informing policy and workforce planning.

## 2. Materials and Methods

### 2.1. Study Design

This study employed a longitudinal observational design and used secondary quantitative data from four annual surveys conducted between 2021 and 2024. The secondary data were initially collected for purposes unrelated to this study, and all identifying information was removed before the data were made accessible to the research team. The Institutional Review Board of Chung Shan Medical University Hospital, Taiwan, reviewed and approved the study protocol on 24 January 2025 (CSMUH No: CS1-24226). All research procedures adhered to ethical guidelines, ensuring confidentiality, anonymity, and proper handling of the secondary data throughout the study.

### 2.2. Participants and Data Collection

The data were collected from a hospital affiliated with a medical university in Taichung, Taiwan. The questionnaires were distributed via QR code-linked emails to staff employed for over a year. To minimize potential non-response bias, we adopted a full-population recruitment strategy rather than sampling. Although we did not conduct a formal comparison between respondents and non-respondents, the institutional design of annual universal invitations and voluntary participation helped to reduce systematic exclusion. Nevertheless, we acknowledge that some degree of non-response bias cannot be entirely ruled out. After applying the exclusion criteria, a total of 1155 participants were included in the final longitudinal analysis.

### 2.3. Measures

All questionnaires included participants’ basic demographic information, living habits, occupational characteristics, and the Copenhagen Burnout Inventory (CBI). Developed by researchers in Denmark, the CBI is recognized for its exceptionally high internal reliability, with Cronbach’s alphas reaching 0.85–0.87. It was designed to be both comprehensible and accessible to all individuals [26]. We used the traditional Chinese version of the CBI [27] to align with the national context. We ensured its applicability to all participants by adopting the personal burnout (PB) scale from the CBI, a universal tool for measuring fatigue or exhaustion levels regardless of occupational status, including staff who do not face clients [26]. The six questions for the PB scale are listed in Supplementary Table S1. The response options were “always”, “often”, “sometimes”, “seldom”, and “never/rarely”, which were assigned scores of 100, 75, 50, 25, and 0, respectively. The mean score of the six items was used as an index of the PB level. The CBI was not a diagnostic criterion for burnout; therefore, we defined the upper quartile of the mean PB score from the first wave in 2021 as the criterion for determining high PB levels (HPBL) in subsequent waves from 2022 to 2024. This cutoff approach is commonly used in studies utilizing the Copenhagen Burnout Inventory (CBI), particularly when clinical diagnostic thresholds are unavailable. Additionally, the upper-quartile threshold allowed us to categorize participants dichotomously, which was necessary for conducting survival analysis based on time-to-event outcomes.

### 2.4. Variable Definitions

The response options for sex were “female” and “male”, and participants reported their age in years. Marital status was categorized as “married” or “other”. Participants’ height and weight were measured, and BMI was reclassified into four groups per the criteria established by the Health Promotion Administration, Ministry of Health and Welfare, Taiwan: underweight (BMI < 18.5), healthy weight (18.5 ≤ BMI < 24.0), overweight (24.0 ≤ BMI < 27.0), and obese (BMI ≥ 27.0). The response options for the highest education degree were “PhD”, “Master”, “Bachelor”, and “other”. For overtime, the response options were “Rarely overtime”, “Monthly overtime less than 45 h”, “Monthly overtime between 45 and 80 h”, and “Monthly overtime over 80 h”. In terms of shift work, the questionnaire included the options “irregular”, “regular”, “night”, and “day” shift work. The study also assessed participants’ average sleeping time during work periods, with response options of “less than 5 h”, “5–6 h”, “6–7 h”, “7–8 h”, and “over 8 h”. Whether participants engaged in leisure activities with family and friends (LAFF) during vacations was also surveyed, with the response options of “Never”, “Rarely”, “Occasionally”, “Often”, and “Always”. Participants were asked to select from the chronic diseases listed in the questionnaire; those having one or more were categorized as “suffering from chronic disease”. The response options for professional fields were nurses, administrative staff, physicians (including attending physicians, residents, and nurse practitioners), and technical staff.

### 2.5. Statistical Analysis

The numbers of questionnaire responses received in 2021, 2022, 2023, and 2024 were 1615, 1694, 1608, and 1221, respectively. We excluded participants who sustained HPBL in the first survey or only completed the survey once. All participants were classified by one of the following three outcomes during follow-up: meeting the HPBL criteria, not meeting the HPBL criteria, or being lost to follow-up. Participants who met the HPBL criteria (event = 1) would no longer contribute additional records, and the total duration of observation (in years) would be calculated up to that point. Regarding loss to follow-up, we would adopt the “carrying forward the last observation” [28] method. Thus, the last recorded data and condition before the loss to follow-up would be considered as “not meeting HPBL” (alive; event = 0) and serve as the duration’s endpoint. For participants still “alive” at the end of the study period (2024), the “event” would also be set to zero and would serve as the endpoint for the duration of the study. To address missing data caused by loss to follow-up, we adopted the “last observation carried forward” (LOCF) method, which allowed us to retain participants with incomplete follow-up and preserve their time-to-event records. Participants who provided only one wave of data were excluded from the longitudinal analysis. No multiple imputation was applied, due to the structure of the secondary dataset.

We examined the impact of time on burnout by employing survival analysis [29] to evaluate the occurrence of HPBL during the observation period among the cohort. The Kaplan–Meier method [30] was used to graphically represent endpoint occurrences over time and estimate the survival probability of HPBL in the population under investigation [31]. Survival analyses typically have greater statistical power to detect significant treatment or exposure effects than binary logistic regression, as they take into account the time to an event [30]. Additionally, we performed a stratified analysis of overtime work to examine differences in the survival probability of HPBL using the Log-rank test. Based on previous research that employed logistic regression to examine associations between work-related stressors and burnout outcomes [32,33], we applied a logistic regression model in this study to assess the impact of overtime on the likelihood of developing high personal burnout levels (HPBL), while adjusting for relevant covariates.

Hypothesis 1 (H1) and Hypothesis 2 (H2): were tested in four steps:

Step 1: Descriptive statistics summarized the participants’ basic demographic characteristics and surveyed variables.

Step 2: Survival analysis examined whether the risk of burnout increased over time (H1).

Step 3: Stratified survival analysis evaluated whether experiencing overtime contributed to a higher risk of worsening burnout over time (H2).

Step 4: Multiple logistic regression analysis assessed whether overtime is an independent risk factor for burnout.

The present study used SAS Enterprise Guide 7.1 software (SAS Institute Inc., Cary, NC, USA) for data analysis, and statistical significance was set at *p* < 0.05.

## 3. Results

### 3.1. Descriptive Analysis

Table 1 summarizes the number of individuals who completed the questionnaires between 2021 and 2024 and the PB mean scores’ quartiles (Q1, Q2, Q3). The Cronbach’s alpha for the PB scale was 0.92, 0.93, 0.94, and 0.93 for 2021, 2022, 2023, and 2024, respectively. A PB score above 45.83 (Q3) was defined as HPBL, according to the upper-quartile threshold in 2021. The proportions of individuals classified as exhibiting HPBL were 489 (30.28%) in 2021, 564 (33.29%) in 2022, 591 (36.75%) in 2023, and 397 (32.51%) in 2024. Statistical analysis showed significant variation in the mean PB scores across the years (*p* = 0.008), with the highest mean recorded in 2023.

### 3.2. Longitudinal Observation and Attrition

**Figure 1:**
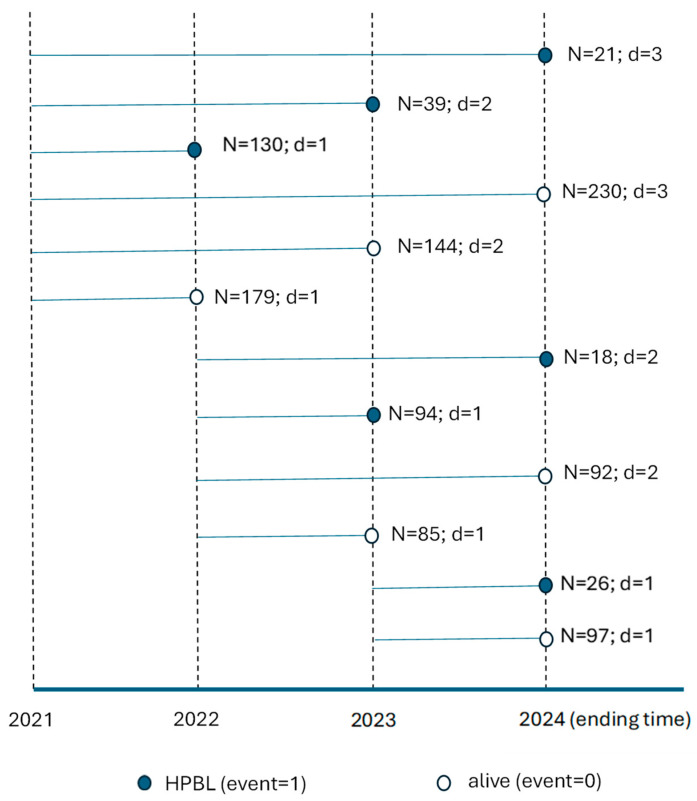
The results of the follow-up from 2021 to 2024. N, individuals; d, duration between the starting and ending follow-up.

Figure 1 illustrates the observation process, excluding individuals who either met the HPBL criteria for the first time or were lost to follow-up by the second year. The final observation cohorts comprised 743 participants in 2021, 289 in 2022, and 123 in 2023.

Among the observation cohorts from 2021, 21 individuals met the HPBL criteria in 2022, 39 in 2023, and 130 in 2024. Additionally, 179 individuals in 2022 and 144 in 2023 did not meet the HPBL criteria, but were subsequently lost to observation the following year. In 2024, the observation of 230 individuals who did not meet the HPBL criteria was concluded.

Among the observation cohorts from 2022, 94 individuals met the HPBL criteria in 2023 and 18 in 2024. Additionally, 85 individuals did not meet the HPBL criteria in 2023 but were subsequently lost to observation the following year. In 2024, the observation of 92 individuals who did not meet the HPBL criteria was concluded.

In 2024, 26 individuals from the 2023 observation cohorts met the HPBL criteria, whereas 97 individuals did not in the same year.

Table 2 summarizes the demographic variables and observation results for the 1155 participants who completed the observation. A total of 358 (48.18%) individuals in 2021 and 85 (29.41%) in 2022 were lost to follow-up. The participant mean age of those who continued observation was 40.66 ± 10.31 years in 2021, 37.07 ± 10.17 years in 2022, and 36.15 ± 10.57 years in 2023. The participants were predominantly female, accounting for 74.80–85.81% of the cohorts, among whom the proportion that met the HPBL criteria (event = 1) ranged from 21.14% to 38.75%.

The mean duration of observation was 1.92 ± 0.87 years in 2021, 1.38 ± 0.49 years in 2022, and 1.21 ± 0.41 years in 2023. The number of participants holding a Master’s or PhD degree was 144 (19.38%) in 2021, 51 (17.65%) in 2022, and 26 (21.14%) in 2023. Regarding professional fields, the number of physicians and nurses was 59 (7.94%) and 235 (31.63%) in 2021, 30 (10.38%) and 141 (48.79%) in 2022, and 14 (11.38%) and 37 (30.08%) in 2023, respectively.

The symbols “+/−” represent the state change between the last and previous observations. Specifically:

“Getting married+” indicates that the participant reported being newly married in the most recent observation survey.

“LAFF+” represents an increase in the frequency of leisure activities with family and friends (LAFF) compared to the previous survey.

“Overtime+” indicates an increased frequency of overtime compared to the previous survey.

“Sleep time−” represents a decrease in sleep time compared to the previous survey.

“Shift work+” indicates transitioning from a regular day/night schedule to shift work.

“OW/OB+” signifies that the participant’s body weight changed from being underweight or a healthy weight to being overweight or obese in the final observation.

“Chronic diseases+” indicates that the participant began to suffer from at least one chronic disease in the final observation.

Among the participants in 2021, 2022, and 2023, 20 (2.69%), 8 (2.77%), and 5 (4.07%) individuals, respectively, reported being newly married. A relative increase in the frequency of leisure activities with family and friends (LAFF) over the previous survey was observed in 149 (20.50%), 56 (19.38%), and 31 (25.20%) participants, respectively. Regarding overtime status, 84 (11.31%), 48 (16.61%), and 20 (16.26%) individuals reported transitioning from rarely working overtime to working over 45 h in overtime monthly. Furthermore, 191 (25.71%), 55 (19.03%), and 33 (26.83%) participants reported sleeping less during their working periods.

In terms of work schedules, 35 (4.71%), 23 (7.96%), and 9 (7.32%) individuals reported shifting from day/night work to shift work. Twenty-two (2.96%), 13 (4.50%), and 4 (3.25%) participants increased from being underweight/a healthy weight to overweight/obese. Additionally, 65 (8.75%), 36 (12.46%), and 9 (7.32%) participants recently reported being diagnosed with at least one chronic disease.

### 3.3. Survival Analysis

**Figure 2:**
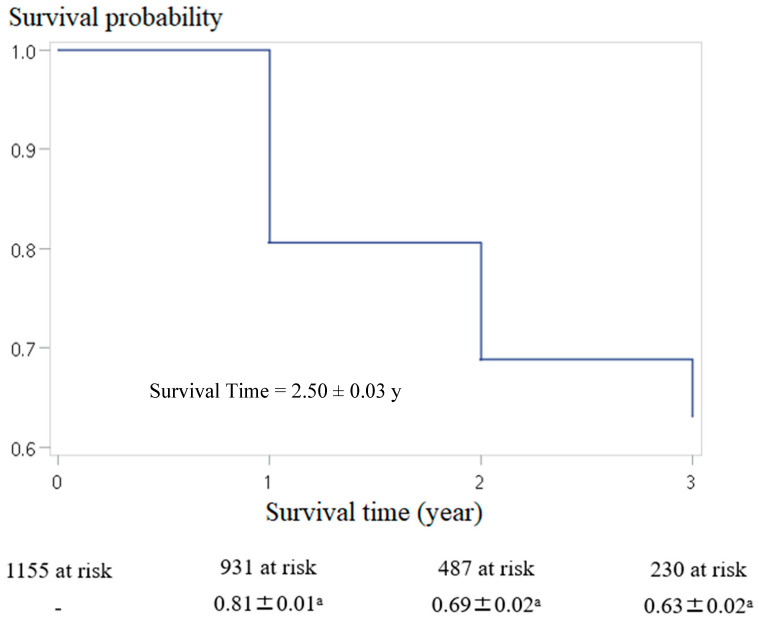
Survival analysis of HPBL. ^a^, mean ± standard error for survival probability.

Figure 2 illustrates the survival probability over time of the participants with HPBL, with the mean survival time estimated at 2.50 ± 0.03 years. After one year, the survival probability was 0.81 ± 0.01, with 931 participants remaining at risk. By the second year, the survival probability decreased to 0.69 ± 0.02 (487 participants at risk), and by the third year, it further declined to 0.63 ± 0.02 (230 participants at risk). All survival probabilities were calculated using Kaplan–Meier estimates. Thus, Hypothesis H1 about the risk of burnout increasing over time is confirmed.

**Figure 3:**
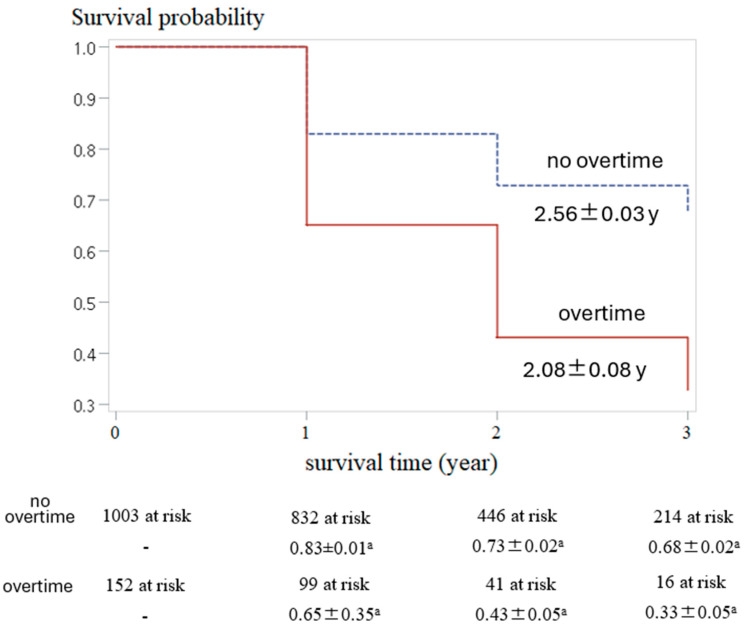
Stratified analysis for the survival analysis of HPBL. ^a^, mean ± standard error for survival probability.

Figure 3 demonstrates that individuals who work overtime have significantly shorter survival times for HPBL and higher probabilities of HPBL progressing than those who do not work overtime. The mean survival time was 2.56 ± 0.03 years for individuals who did not work overtime, compared to 2.08 ± 0.08 years for those who did (Log-rank test, *p* < 0.0001). The probability of survival was 0.83 and 0.65 at year 1, 0.74 and 0.43 at year 2, and 0.68 and 0.33 at year 3 for the no-overtime and overtime groups, respectively (Log-rank test, *p* < 0.0001). These results provide strong evidence in support of Hypothesis H_2_ that individuals who work overtime are at a significantly higher risk of burnout that worsens over time.

### 3.4. Logistic Regression Analysis

M_0_ in Table 3 identifies potential risk factors for high personal burnout levels (HPBL), including overtime (+), age, female gender, being a physician or nurse, and reduced sleep time (−). These variables were subsequently included as covariates in the multivariate logistic regression model (M_1_). The results demonstrate that overtime, female gender, being a physician or nurse, and reduced sleep time are significant independent risk factors for HPBL. To assess the potential impact of multicollinearity among predictors, a variance inflation factor (VIF) analysis was conducted using a multiple linear regression model that included all covariates. The results showed no substantial multicollinearity, with all VIF values below the conventional threshold of 5. Detailed results are presented in Supplementary Table S2.

Specifically, individuals who reported working overtime in the past year had significantly higher odds of experiencing HPBL compared to those who rarely worked overtime (OR = 3.14, *p* < 0.001), thus supporting Hypothesis H_2_. Female employees had a higher likelihood of HPBL than their male counterparts (OR = 1.74, *p* = 0.008). In terms of professional role, physicians (OR = 1.94, *p* = 0.008) and nurses (OR = 1.75, *p* = 0.001) exhibited significantly greater odds of HPBL than other healthcare workers. Furthermore, participants who reported a reduction in daily sleep duration compared to the previous year had a 40% increased likelihood of developing HPBL (OR = 1.40, *p* = 0.029).

## 4. Discussion

In response to the threat of COVID-19, hospitals across Taiwan adopted various epidemic prevention measures, including daily temperature and health monitoring for all staff, service recipients, and accompanying persons, starting on 7 June 2021. These measures were gradually lifted from May 2023 onwards [34,35]. As shown in Table 1, healthcare workers reported higher levels of PB in 2022 and 2023 than in 2021 and 2024, aligning with the prolonged challenges during the pandemic period of COVID-19. These trends may reflect not only overtime-related organizational stress, but also cumulative psychological strain from pandemic disruptions. While COVID-19 was not the primary variable of interest, its potential confounding influence should be acknowledged and warrants more detailed exploration in future studies.

Our longitudinal observational study of healthcare workers found that the risk of PB increases over time (H_1_) and that individuals who work overtime face a higher risk of worsening burnout than those who do not work overtime (H_2_). These findings support our proposed hypotheses.

### 4.1. Burnout’s Progressive Onset

Figure 2 illustrates that the survival probability of HPBL decreases over time, reflecting a significant and progressive worsening of burnout among individuals. This trend aligns with the progressive stages of burnout development [4], which is confirmed by previous studies. For example, research on burnout among frontline physicians in the US during the pandemic demonstrated that fatigue and frustration progressively worsened burnout [25]. Similarly, female residents experienced increased emotional exhaustion between their second and third years of residency [36].

Why does the risk of burnout increase over time? Burnout is widely recognized as a multistage process that typically begins with work-related stress [4]. In the initial phase, stress can diminish enthusiasm and lead to stagnation. Prolonged exposure to stress results in frustration, gradually depleting energy and enthusiasm for work. If left unaddressed, this progression may culminate in significant physical and emotional challenges that require professional intervention [3].

These findings show the gradual nature of burnout development and underscore a critical window for intervention. Early identification of individuals who are at risk and targeted strategies during burnout’s early stages play pivotal roles in mitigating its progression and reducing its long-term consequences.

### 4.2. Risk Factors of Burnout

We have established that burnout worsens over time. Before examining the impact of overtime on burnout, we must first identify and control for potential confounders. One notable factor is gender. The data presented in Table 3 indicate that female employees had a significantly higher risk of HPBL than their male peers during the observation period (HR = 1.49, *p* = 0.025). Consistent with previous findings, female healthcare workers were more vulnerable to burnout, which may relate to work–life imbalance and social expectations (OR = 1.74, *p* = 0.008). This finding is consistent with prior research, which has consistently reported gender-related disparities in HPBL risk. Understanding this disparity is critical because it may reflect underlying differences in work–life balance pressures, societal expectations, or coping mechanisms. These gender-specific factors should be considered in future interventions aimed at mitigating burnout. A cross-sectional survey of members of the American College of Surgeons demonstrated that more women than men surgeons suffered from burnout due to work–home conflicts [37]. Likewise, a longitudinal study found that female residents experienced emotional exhaustion more easily than male residents [36]. These reasons may stem from slower promotions, lower salaries, fewer resources, and inequality, resulting in pressure related to decisions on when to have children, as well as conflict between fulfilling roles as a spouse and parent while pursuing a career [38,39]. Addressing these gender-specific issues is critical for designing interventions that effectively mitigate burnout and promote equity in the workplace. These findings suggest that healthcare organizations should consider gender-specific interventions—such as flexible scheduling, supportive parental leave policies, and leadership development programs for women—to address structural stressors and promote equity.

The prevalence of burnout among physicians in the US is approximately 50%, twice as high as that of the general working population [40]. Similarly, the prevalence of burnout among nurses ranges from 10.51% to 33% worldwide, depending on regional and methodological differences [12,41]. Both physicians and nurses are considered to be high-risk populations for burnout. Physicians and nurses consistently emerged as high-risk groups for burnout in our adjusted models, reflecting occupational demands and chronic stress exposure.

This trend indicates that occupational roles influence burnout risk, likely driven by workload, stress, and workplace challenges. Our findings confirm the classification of physicians and nurses as high-risk groups for burnout. Future research should identify and address the specific drivers of burnout within these professions to mitigate its impact and enhance the well-being of healthcare workers.

Sleep duration has been linked with mortality [42] and is increasingly recognized as a critical factor in burnout. Research studies have consistently identified insufficient sleep, defined as less than 6 h per night, as a significant risk factor for burnout [43]. Consistent with these findings, sleep deprivation, already recognized as a physiological stressor, was confirmed to be closely linked with burnout in our analysis. These results highlight the critical role of adequate sleep in mitigating burnout risk, and suggest that interventions targeting sleep hygiene may effectively reduce burnout prevalence among high-risk populations. Given that sleep deprivation was associated with a 40% increased risk of HPBL, institutions should implement safeguards such as regulated duty hours, mandatory rest periods, and routine fatigue monitoring, particularly in shift-based roles.

Previous cross-sectional studies have demonstrated that higher levels of burnout are associated with increased fat intake [44] and a higher risk of obesity [45], suggesting a possible bidirectional relationship between burnout and body weight. In our study, logistic regression analysis showed that being overweight or obese was marginally associated with a higher risk of high personal burnout level (HPBL) (OR = 1.79, *p* = 0.079). Although this association did not reach conventional statistical significance, the trend is consistent with prior findings and implies that weight management may play a role in mitigating burnout risk. Further research is warranted to clarify this relationship. Although the association did not reach conventional significance, the observed trend supports integrated wellness strategies that target both physical and psychological health, such as workplace fitness programs, nutrition counseling, and burnout screening.

Nevertheless, the causal relationship between obesity and burnout remains unclear. Longitudinal studies are needed to determine whether obesity predisposes individuals to burnout or whether burnout contributes to obesity. These findings emphasize the importance of addressing obesity as part of a comprehensive strategy to mitigate burnout, especially in high-risk occupational groups.

### 4.3. The Effect of Overtime on Burnout

Overtime work poses a substantial risk to workers’ mental health. A study conducted in Japan found that extended overtime hours were significantly associated with heightened levels of anxiety and depression [18]. Similarly, involuntary overtime among nurses has been shown to adversely impact mental health and work engagement [46]. Building on this evidence, several cross-sectional studies have established a correlation between overtime work and burnout [47,48]. However, cross-sectional designs limit the ability to establish causal relationships, suggesting the need for longitudinal research to investigate the temporal effects of overtime on burnout.

We adopted survival and Cox regression analyses to address this limitation and explore the relationship between overtime and burnout. These methods provided evidence supporting a causal link between increased overtime work and burnout. Specifically, individuals who reported an increased frequency of working overtime in the past year had significantly shorter survival times and a greater risk of HPBL (Figure 3 and Table 3, M1). These findings underscore the critical role of overtime in developing burnout and highlight the necessity for targeted interventions to mitigate this risk, such as workload management and policies to limit excessive overtime.

Finally, we propose a possible explanation for the relationship between overtime work and burnout through the lens of work–family conflict. Overtime work often exacerbates work–family conflict [49], primarily by impeding psychological detachment from work [50]. Drawing on the 12-stage model of burnout development [4], we hypothesize that individuals initially approach work with enthusiasm. As work demands grow, they may begin to neglect their personal needs and relationships, which may escalate unresolved conflicts, shift personal values, and lead to denial of existing problems, ultimately contributing to burnout.

While this hypothesis provides a theoretical framework for understanding the link between overtime and burnout, additional empirical research is needed to validate these mechanisms. Future interventions could focus on promoting work–life balance and supporting psychological detachment from work, mitigating the effect of overtime work on burnout.

These findings must be interpreted within the specific organizational and cultural context of Taiwan’s healthcare system. Asian healthcare institutions are often characterized by strong hierarchical structures and collectivist values that prioritize obedience and endurance over self-expression or psychological disclosure [51,52]. This may discourage help-seeking behaviors, even in the face of emotional exhaustion. In Taiwan, physicians and nurses are frequently subject to long work hours and systemic overtime, which are perceived as normative and often go unchallenged [14,53]. Compared to Western systems—where psychological support, work–life balance, and burnout prevention strategies are more institutionalized—these contextual features in Asia may contribute to the high prevalence and under-reporting of burnout. Acknowledging such cultural and structural factors is essential for developing contextually appropriate interventions.

To translate these findings into actionable strategies, healthcare institutions should consider implementing evidence-based interventions that address both workload and recovery. For example, rotational staffing systems, task redistribution, and weekly hour limits can help in managing workloads effectively. To enhance psychological detachment, strategies such as “no after-hours email” policies, scheduled recovery breaks, and training in mindfulness and boundary-setting may support mental disengagement from work stress. These organizational-level interventions have been shown to improve employee well-being and reduce burnout risk [54,55,56].

## 5. Limitations

The study encountered a loss-to-follow-up rate exceeding 20% (Table 2, 2021: 48.18%; 2022: 29.41%), which may have introduced bias and limit the generalizability of our findings [57]. A high loss-to-follow-up rate could lead to the under-representation of certain population groups and affect the observed trends’ robustness.

While our results indicate increasing burnout over time, the high loss-to-follow-up rate suggests that caution should be taken in interpreting these findings. Future studies should aim to improve participant retention through enhanced follow-up strategies.

Our research design investigated whether overtime work increased during two periods. Still, this approach ignores the burnout of those who have worked overtime for many years, and cannot determine whether reducing overtime can improve burnout.

Unlike illness or death, burnout is dynamic and may fluctuate between surveys. The 1-year survey interval may have failed to capture transient episodes, potentially overestimating the survival time.

We must emphasize that HPBL is a relatively high value observed within a specific survey population; it is not a clinical diagnostic criterion. Additionally, it may vary across different survey populations. Therefore, to avoid misunderstanding, we should refrain from using terms such as “suffering from burnout” in our conclusions, as this could incorrectly imply a clinical diagnosis. Furthermore, the definition of HPBL based on the upper quartile of the 2021 baseline PB score reflects a relative, sample-specific threshold that lacks external validity. While this approach was appropriate for capturing intra-cohort variation and tracking burnout progression within the same population, it may limit the generalizability of our findings to other populations or clinical settings. Currently, no universally accepted cutoff values for the Copenhagen Burnout Inventory (CBI) have been established. Future research should consider applying population-based normative values or validated clinical thresholds, where available, to enhance comparability and external applicability.

Another limitation of this study concerns the handling of loss to follow-up, which exceeded 20% in certain cohorts. To address this, we adopted the “last observation carried forward” (LOCF) approach, which allowed us to retain participants with partial follow-up and maintain consistency in survival time calculations. However, this method assumes that a participant’s status remains unchanged after their last recorded observation, which may not adequately capture dynamic psychological processes such as burnout. This assumption could potentially lead to biased estimates of time-to-event outcomes or attenuate observed associations. While the use of LOCF was necessitated by the structure of our secondary dataset, we acknowledge that more advanced imputation techniques (e.g., multiple imputation or joint modeling) or sensitivity analyses would offer more robust treatment of missing data. Future studies should consider applying such approaches when data availability and structure permit.

Although we ultimately replaced the Cox regression with logistic regression due to violation of the proportional hazards assumption, we acknowledge the importance of formally testing this assumption. The bootstrapped maximum absolute value test revealed significant violations for several baseline variables, including gender, overtime, and professional role (Supplementary Table S3), thereby supporting our methodological decision.

## 6. Conclusions

This study identifies several significant predictors of burnout among healthcare workers, including female gender, professional role (physician or nurse), and reduced sleep duration. Notably, overtime work emerged as a key independent risk factor, with effects that appear to accumulate over time. These findings reinforce the understanding of burnout as a progressive condition rather than one with a sudden onset, underscoring the importance of early detection and timely intervention.

From a practical perspective, healthcare institutions should implement targeted strategies to mitigate burnout risk. Given the higher burnout risk observed among female staff and clinical professionals, recommended measures include gender-sensitive workload planning, stress management programs, and psychological counseling tailored to high-risk groups. In response to the observed association between reduced sleep and burnout, fatigue risk management and protected rest policies should be considered. Because overtime was identified as the strongest predictor, institutions are encouraged to adopt real-time overtime monitoring systems and enforce monthly limits.

Such efforts may not only reduce burnout prevalence, but also enhance employee well-being, workforce retention, and the overall quality of healthcare delivery.

Academically, this study advances the literature by applying a longitudinal design to examine the temporal dynamics of burnout onset, moving beyond cross-sectional prevalence estimates. Furthermore, it situates the findings within the distinct organizational culture of Taiwanese healthcare settings, contributing culturally grounded insights to an area that remains underexplored. These methodological and contextual strengths distinguish the present study and provide a robust foundation for future research and intervention development.

## Figures and Tables

**Table 1:** Personal burnout score for all individuals (2021–2024).

	2021	2022	2023	2024	
N	1615	1694	1608	1221	*p*
α	0.92	0.93	0.94	0.93	
mean ± SD	36.07 ± 18.05 ^a^	37.14 ± 18.66 ^ab^	38.19 ± 19.83 ^b^	36.32 ± 18.67 ^a^	0.008
Q1	25.00	25.00	25.00	25.00	
Q2	33.33	33.33	37.50	33.33	
Q3	45.83 ^+^	50.00	50.00	50.00	
HPBL					
N	489	564	591	397	
%	30.28	33.29	36.75	32.51	

N, individuals; SD, standard deviation; α, Cronbach’s alpha for PB scale; %, proportion; ^+^, judgment criteria for HPBL; P, *p*-value from one-way ANOVA; ^a,b^, means with the same letter are not significantly different (by Duncan’s multiple-range test).

**Table 2:** The basic demographic and surveyed variables for the participants who completed follow-up.

	Mean ± SD/Individuals (%)		
Surveyed Variable	2021 (N = 743)	2022 (N = 289)	2023 (N = 123)
Lost to follow-up	358 (48.18)	85 (29.41)	-
Age (years)	40.66 ± 10.31	37.07 ± 10.17	36.15 ± 10.57
Female	613 (82.50)	248 (85.81)	92 (74.80)
HPBL (event = 1)	190 (25.57)	112 (38.75)	26 (21.14)
Duration (year)	1.92 ± 0.87	1.38 ± 0.49	1.21 ± 0.41
Master’s or PhD	144 (19.38)	51 (17.65)	26 (21.14)
Physicians	59 (7.94)	30 (10.38)	14 (11.38)
Nurses	235 (31.63)	141 (48.79)	37 (30.08)
Technical staff	117 (15.75)	21 (7.27)	23 (18.70)
Administration staff	332 (44.68)	97 (33.56)	49 (39.83)
Getting married+	20 (2.69)	8 (2.77)	5 (4.07)
LAFF+	149 (20.05)	56 (19.38)	31 (25.20)
Overtime+	84 (11.31)	48 (16.61)	20 (16.26)
Sleep time−	191 (25.71)	55 (19.03)	33 (26.83)
Shift work+	35 (4.71)	23 (7.96)	9 (7.32)
OW/OB+	22 (2.96)	13 (4.50)	4 (3.25)
Chronic diseases+	65 (8.75)	36 (12.46)	9 (7.32)

N, individuals; SD, standard deviation.

**Table 3:** The results of the univariate and multivariate logistic regression analyses for HPBL.

	Logistic Regression for HPBL			
	Univariate Analysis (M_0_)		Multivariate Analysis (M_1_)	
Surveyed Variable	OR	*p*	OR	*p*
Overtime (+)	3.28	<0.001	3.14	<0.001
Age	0.99	0.042	1.00	0.676
Female	1.75	0.003	1.74	0.008
Getting married+	1.27	0.524	-	-
LAFF (+)	0.88	0.417	-	-
Master’s or PhD	1.09	0.591	-	-
Physicians	1.90	0.007	1.94	0.008
Nurses	2.04	<0.001	1.75	0.001
Technical staff	1.39	0.118	1.39	0.128
Sleep time (−)	1.46	0.011	1.40	0.029
Shift work (+)	1.16	0.582	-	-
OW/OB (+)	1.79	0.079	-	-
Chronic diseases (+)	0.94	0.783	-	-

OR, odds ratio; *p*, *p*-value; M_0_, simple logistic regression without adjusted variables; M_1_, multiple logistic regression, adjusting for age, female gender, physicians, nurses, technical staff, sleep time (−), and OW/OB (+). Model diagnostics for M_1_ indicated acceptable fit and discrimination: Hosmer–Lemeshow goodness-of-fit test χ^2^ = 13.17, df = 8, *p* = 0.1062; c-statistic (AUC) = 0.652; Akaike Information Criterion (AIC) = 1380.321; −2 Log Likelihood = 1378.321.

## Data Availability

Data are available from the corresponding author upon reasonable request.

## Supplementary Materials

The following supporting information can be downloaded at https://www.mdpi.com/article/10.3390/healthcare13151859/s1: Table S1: Items of the personal burnout (PB) scale from the Copenhagen Burnout Inventory (CBI); Table S2: Multicollinearity diagnostics based on multiple linear regression for predictors of HPBL; Table S3: Test of proportional hazards assumption (PH) using bootstrapped maximum absolute value method. Refs. [58,59] are cited in the Supplementary Materials.

## Abbreviations

CBI	Copenhagen Burnout Inventory
HPBL	High PB level
LAFF	Leisure activities with family and friends
PB	Personal burnout
