# Supplementary material for: Prolonged Overtime Predicts Worsening Burnout Among Healthcare Workers: A 4-Year Longitudinal Study in Taiwan
Source: Healthcare (Basel). 2025 Jul 30;13(15):1859. doi: 10.3390/healthcare13151859 (PMC12345989; doi:10.3390/healthcare13151859)
Supplement: Supplementary file 1 [file healthcare-13-01859-s001.zip › healthcare-3745818-supplementary.pdf]

Supplementary Table S1 Personal Burnout (PB) Items from the Copenhagen Burnout Inventory (CBI)

**Item Code**	**Item Content**
1	How often do you feel tired?
2	How often are you physically exhausted?
3	How often are you emotionally exhausted?
4	How often do you think “I can’t take it anymore”?
5	How often do you feel worn out?
6	How often do you feel weak and susceptible to illness?

Note: These items are part of the Personal Burnout (PB) domain of the Copenhagen Burnout Inventory (CBI).

Supplementary Table S2 Multicollinearity Diagnostics Based on Multiple Linear Regression for Predictors of HPBL

	Multiple regression for HPBL		
Surveyed variable	B	P	VIF
Overtime (+)	0.26	<.001	1.015
Age	-0.00	0.667	1.077
Female	0.09	0.010	1.125
Physicians	0.12	0.012	1.151
Nurses	0.11	<.001	1.339
Technique staff	0.06	0.152	1.163
Sleep time (−)	0.07	0.030	1.005

B, linear regression coefficient; VIF, variance inflation factor

Supplementary Table S3 Test of Proportional Hazards Assumption (PH) Using Bootstrapped Maximum Absolute Value Method

Variable	Max Abs Value	Reps	Seed	P-value (Pr > MaxAbsVal)
Age	11.3079	1000	1713534637	0.2620
Female	0.0000	1000	1713534637	<0.0001
OW_OB (+)	0.0010	1000	1713534637	<0.0001
Sleep_time (−)	0.0004	1000	1713534637	<0.0001
Overtime (+)	0.0041	1000	1713534637	<0.0001
Physicians	0.0001	1000	1713534637	<0.0001
Nurses	0.0001	1000	1713534637	<0.0001
Technique staff	0.0001	1000	1713534637	<0.0001

Note: Max Abs Value = Maximum Absolute Value of the test statistic derived from bootstrapped residuals. P-values < 0.05 indicate significant violation of the proportional hazards assumption. The testing method follows the frameworks described by Lin et al. (1993) and Grambsch and Therneau (1994).

References:

1. Grambsch, P.M.; Therneau, T.M. Proportional hazards tests and diagnostics based on weighted residuals. *Biometrika* **1994**, *81*(3), 515–526. https://doi.org/10.1093/biomet/81.3.515
2. Lin, D.Y.; Wei, L.J.; Ying, Z. Checking the Cox model with cumulative sums of martingale-based residuals. *Biometrika* **1993**, *80*(3), 557–572. https://doi.org/10.1093/biomet/80.3.557
